# Time of Care and Time of Dying: A Multidisciplinary Case Report on End-of-Life Experience Within the Italian Legal Framework

**DOI:** 10.3390/healthcare13212741

**Published:** 2025-10-29

**Authors:** Letizia Iannopollo, Eleonora Pinto, Pamela Iannizzi, Flavia Salmaso, Alessandra Feltrin

**Affiliations:** 1 Hospital Psychology, Veneto Institute of Oncology IOV-IRCCS, 35128 Padua, Italy; letianno72@gmail.com (L.I.); pamela.iannizzi@iov.veneto.it (P.I.); alessandra.feltrin@iov.veneto.it (A.F.); 2 Palliative Care Unit, IRCCS Istituto Oncologico Veneto IOV, 31033 Castelfranco Veneto, Italy

**Keywords:** palliative care, end-of-life ethics, multidisciplinary team, patient self-determination, dignity therapy

## Abstract

In this segment of the Palliative Care Unit case series, we introduce a patient with a long history of oncological treatments for recurrent breast cancer. After active treatments and a global control of the neoplasm, disease progression made the patient access different lines of chemotherapies, even asking for them in anticipation of a few advantages in the balance between benefits and risks. When the patient decided to permanently discontinue chemotherapy, she felt she had disrupted her values. Also, as a reaction to breaking bad news without estimating alternative paths, she considered her deteriorating condition as the sole criterion for assisted dying in another country. Could this be a self-consistent choice for this patient, so determined to find and pursue possibilities in treatment previously? Should this clue respond precisely to the patient’s needs? This contribution’s objective is to debate possibilities of patient self-determination and dignity at the end of life by integrating psychological support, palliative care, and legal–ethical awareness. This case study presents multidisciplinary team work through some key turning points. This team work was carried out in a national context that is currently inconclusive regarding assisted suicide, since active euthanasia is illegal. At the same time, the national Constitutional Court (242/2019) recently opened the possibility of eventual medically assisted suicide under certain circumstances. In this case, health professionals considered this context and tried to delve deeply into respecting the patient’s identity in order to determine when and if the exceptional circumstances were met. This case highlights the ethical sense of end-of-life accompaniment, which when conducted by physicians, nurses, and psychologists together can lead to effective support and allow patients to maintain their identity and to express themselves respecting not only their fears but also their vision of themselves as human beings. A first key turning point was, for instance, taking into account the patient’s history and values, and a subsequent one was supporting the patient in exploring healthcare services and related end-of-life support. In a further key turning point, the patient was helped in engaging with physicians in order to understand types of continuous care, as well as the timing and expected results of sedation. Finally, she chose a healthcare service where she could spend the end of her life in fulfillment of her values. Overall, this case report illustrates how integrating psychological support, palliative care, and legal–ethical awareness can promote patient self-determination and dignity at the end of life.

## 1. Introduction

Helping patients in managing end-of-life choices management, thus facilitating access to comprehensive care, is the core of this case report. Reflection on an oncological pathway and the integrated psycho-oncological care provided emerges from the events described in the course of treatment presented. Such reflection suggests the need for coordinated action by the multidisciplinary team and a comprehensive caretaking model for dignity in dying.

From a regulatory point of view, the main national references for end-of-life legislation are the Italian law [1,2,3] on informed consent, the Constitutional Court Judgments [4,5,6], and the Code of Medical Ethics [7]. The clinical case described here concerns a national context currently that remains inconclusive regarding assisted suicide [8]. Indeed, the request for the affirmation of a right to euthanasia has been rejected, and the facilitation of suicide in the Italian system is not possible as a matter of principle. At the same time, the national Constitutional Court [4] recently opened the possibility of eventual medically assisted suicide under certain circumstances. Exceptionally, medically assisted suicide can be supported when three conditions are met: an irreversible disease that is a source of serious suffering; the dependence of the patient on vital supports; and the full and expressed will of the patient to end their life. Subsequently, the procedure that must be undertaken at this point involves various phases, and the patient must formally contact the National Health Service so that the presence of the requirements is verified. A specific commission verifies the validity of the application and draws up and sends a report to the territorially competent ethics committee. The patient must also be informed about possible alternative solutions, such as access to palliative care. Then, the ethics committee gives its opinion: only after this step can the assisted suicide be authorized. But the entire procedure can take years.

Therefore, the objective of this case report is to illustrate how multidisciplinary care, including psychological and palliative interventions, may shape a patient’s end-of-life experience and decision-making process within the Italian legal framework.

## 2. Case Description

In 2012, Mrs. A. was diagnosed with breast cancer and began neoadjuvant chemotherapy followed by a mastectomy. Over the following decade, her care trajectory evolved through multiple lines of active therapy, culminating in intensive palliative care and hospice admissions. Details are presented in Supplementary Material.

The diagnosis of a breast neoplasm is made at a general hospital, where the patient, Mrs. A., undergoes neoadjuvant chemotherapy followed by a mastectomy. Finally, she starts hormone therapy. Later, peritoneal and ovarian metastases are diagnosed, so the patient decides to seek care at a tertiary referral hospital, where she starts a new line of chemotherapy, followed by maintenance therapy. There is overall control of the disease for 9 years, but an ovarian recurrence of the mammary tumor and several metastatic peritoneal nodules are detected.

Later that year, the patient is invited by her oncologist to see a psychologist for anxiety and demoralization, and this care pathway—during which informed consent for treatment and for scientific research is obtained—continues throughout the entire medical course of treatment. Mrs. A. and the psychologist agree on her inclusion in group psychotherapy, partly to enable the patient to rebuild a social support network that is lacking at that time. The patient participates in different types of group treatment, with emotional, narrative, and cognitive focus.

Three years later (2022), additional peritoneal disease progression makes the patient access an experimental clinical trial. In the same year, her genetic analysis reveals BRCA2-positive results. As a consequence of increasing fatigue and asthenia, decreased performance status, drug-related peripheral neuropathy, and anxiety symptoms, simultaneous palliative care is initiated, and active therapies are temporarily stopped due to tumor stability.

In 2023, a new progression of disease with peritoneal involvement occurs and chemotherapy treatments are resumed, but at the beginning of 2024, because of deterioration in her general conditions with asthenia and limited pain control, the patient increases her visits to a palliative care physician, who also notes a worsening of the patient’s mood. So, after hospitalization in a general medical ward, the experimental therapy is stopped. When a further peritoneal progression of the disease is diagnosed, the oncologist explains the clinical situation, prognosis, and treatment options to the patient, and they decide to start a new line of treatment, well aware of the reduced chances of cancer control.

At the beginning of 2025, the patient is informed by her oncologist the worsening of her clinical status, and after a discussion on possible treatment options, highlighting the limited benefits expected and the risks due to side effects, Mrs. A. decides to maintain active therapy and home care, continuing the therapy one more time, with an intensification of simultaneous palliative care at home.

Then, she suddenly shifts from active therapy to hospice: after a few weeks, due to ongoing care needs for frequent drainage, vomiting, and poor nutrition, the patient decides to definitively stop chemotherapy and requests admission to a hospice in order to receive continuous care. In the meantime, psychological support is provided via telemedicine during the last months of the patient’s life, addressing the difficulties she expresses until hospice admission. During her stay, one week after hospice admission, the patient learns about the possibility of palliative sedation and consents to it, until her occurs in a manner reported to be peaceful.

Accordingly, the overall duration of the treatment is about 13 years, of which 2 years involve simultaneous and palliative care. 

## 3. The Role and Meaning of Palliative Treatments in End-of-Life Care

The WHO definition of palliative care introduces the meaning of care as a process to be built with patients, for them and their families, rather than considering it as something to which the patient must conform in an impersonal and undeniable way [8,9]. In 2025, a few weeks after Mrs. A. had decided to undergo further treatment, she decided to stop chemotherapy definitively due to her ongoing supportive care needs. Could this have been a self-consistent choice for this patient, who was previously so determined to find and pursue every possible treatment option? Should this clue be understood as a precise response to the patient’s needs?

It was likely a suspension of the patient’s identity and sense of agency. Through the long psychological support, the specialist could observe that the awareness gained from open communication with the oncologist meant that the patient knew that once the treatment was suspended, she would soon die. Thus, the time remaining for care was equal to the lifetime. The anticonservative ideation was identified by Mrs. A. as the sole possibility for facing the tumor and intervening: if treatments could not go on, then life should be stopped.

During the treatments, the manner (“how”) and the choice to exercise a right in one way or another, or not to exercise it, also reflect on dying as the result of a series of choices. Therefore, the choices made when the patient has already had the opportunity to learn about illness and its predictable course become particularly important from a legal standpoint. Rooted in the individual right to health, the principle of self-determination in case law is inviolable, and it is an expression of the values of human dignity and personal identity, not only as a subjective right but also as part of the European constitutional heritage [10]. For this reason, throughout the different stages of treatment, the ethical principle followed by the psychologist was to allow the patient to find an active role in evaluating, reflecting on, discussing, and influencing the possible options in care. Indeed, in palliative care decision-making, the patient–healthcare dyad can overwhelm the “no choice” situation [11]. This was also achieved by providing exhaustive and complete information, which made the patient feel empowered. Communication with the palliative care specialist, particularly from a pharmacological point of view, allowed the patient to understand and share medical–technical choices [12].

### 3.1. From “Time of Cure” to “Care Time”

During palliative care, three critical moments of choice arose. Based on these moments of choice, healthcare professionals identified some questions, the answers to which let them create a proper “care time” that was self-consistent for Mrs. A.

#### 3.1.1. Care Time Can Be Directed by the Patient’s Reported Experience

The first critical moment concerned the patient’s realization of her inability to manage symptoms alone, highlighting the importance of timely professional guidance in home palliative care. The patient understood that she could not face everything alone and that she had to necessarily change her strategies if she wanted to achieve symptom improvement. After the transition to home care, the patient experienced suspicion and feelings of defeat, but she neglected them. Due to her psychological characteristics, she needed professional guidance rather than a formal caregiver—someone capable of reassuring her, even just by listening and confirming the suitability of her choices. Indeed, living alone, she monitored and assessed her needs independently, making her own requests for assistance, but without being able to access consultations with specialists with the same regularity she had experienced during active cancer treatment, Mrs. A. encountered many difficulties in the transition from hospital care to home palliative care, including experience of loneliness that fostered fear and uncertainty about her own end of life. The patient also experienced a feeling of abandonment: the experience of “loss”, as reported by the patient, was conditioned by her personal family history; once this aspect was taken into account, she was able to reach a tolerable state that restored meaning to her end-of-life experience. This type of home care organization made the patient feel that her specialists were absent, as she had no physical place to turn to. This aspect appeared to be predominant, making the prescribed psychopharmacological therapies—which she had to use independently as needed—subjectively ineffective. Therefore, Mrs. A. was unable to adapt to caring for herself independently, as she lacked a supportive figure to help her manage the fears triggered by her symptoms [12]. This first critical moment and its management showed that the patient’s experience, when reported, can shape the care time, suggesting possible improvements in the healthcare services for such patients.

#### 3.1.2. Opportunity for Detecting Patient’s Needs

The second critical moment concerned the perceived changes in the different methods of healthcare service provision. This critical moment arose when the patient reported fatigue in managing her relationship with the home care team. During the last months of 2024, following the need to identify effective ways of assisting her with the physical and moral suffering she was experiencing, Mrs. A. was taken into care by a palliative care specialist. After so many years of hospital-based management, the patient imagined finding an organization similar to the hospital. This difficulty required the intervention of a psychologist to give the patient the opportunity to reconcile her palliative care needs with the advantages she perceived in the hospital organization.

On the psychologist’s suggestion, the patient visited the hospice to gain a tangible, concrete sense of the place where she would be cared for, allowing her to familiarize herself with the physical location, the organization of care, and the people and roles of those who would be welcoming her. There, Mrs. A. found the hospitalization regime more similar to the services previously received. She was informed by a hospice physician about the care options with regard to end-of-life choices, comparing them with those offered by home care. Once she was able to identify a place where care was possible, the patient said she could face the end of life without feeling alone. During the last month of life, before entering hospice, the hospital team collaborated as a whole according to Mrs. A.’s requests, including the oncologist, palliative care physicians, psychologist, and general practitioner. This ensured continuity of care, especially from the point of view of the patient’s growing need to be reassured that she would not find herself dying alone. Understanding Mrs. A.’s wish to be able to express her wishes and to die while controlling the painful symptoms and anguish of death also allowed the team to identify the appropriate timing for the pharmacological adjustments. This required continuous information sharing between different healthcare professionals to make clear Mrs. A.’s increasing number of questions reflecting her existential suffering—primarily the fear of pain and anguish of death. Therefore, the multidisciplinary team started planning ethically correct and more effective interventions when Mrs. A.’s psychological difficulties were detected, rather than waiting for symptoms to appear or adapting after secondary diseases had taken their toll. Conversely, choosing a rigid approach would have risked poor timing, which would have led to the patient’s misalignment and increased suffering due to the intolerable inner conflict associated with a sense of inadequacy [13,14,15]. This second critical moment and the related management showed that exploring patients’ needs lets healthcare professionals shape their support and avoid stereotypical mechanisms of support.

#### 3.1.3. A Time for Transition from Patient’s Needs to Solutions of Patient’s Fears

The third critical moment concerned patients’ awareness of changes in cognitive functions. In particular, this critical moment was identified in her distrust of pain therapy: the fear of losing one’s ability to think and also of not being aware of oneself determines an initial limitation to compliance with psychopharmacological and palliative treatment.

A multidisciplinary team, recognizing the phase in which the patient could no longer tolerate the symptoms, took into account the risk of experiencing witness injustice and hermeneutic injustice [16], so that the patient’s credibility would not be lost and her experience was understood. Mrs. A.’s requests were contextualized, in relation to both the stage of disease and the symptoms, particularly when she showed increased anxious–depressive symptomatology, decreased tolerance to frustration, reduced ability to take care of herself, increased social isolation, and increasingly intrusive and distressing thoughts. During pain management, Mrs. A. also experienced episodes of confusion and decreased temporal orientation, with general psycho-physical fatigue. During this critical time, the patient was supported through the adequate time given by care providers [15]. Indeed, she was helped not only to balance the risks and benefits of drug treatment but also to recognize the existential distress arising from the gap between her idealistic expectations and current clinical and pharmacological conditions. As a result, Mrs. A.’s conscious identification of a place for her end-of-life, where a proper meaning was possible, provided immediate relief from her existential distress.

This third moment enabled the healthcare professionals to accept and engage with the patient’s worries and fears instead of avoiding them—allowing them to face them together with the patient.

## 4. Ethics in the Time of Dying

Overall, in the Italian legal framework, “passive euthanasia”, which refers to the suspension of care allowing the patient to refuse any type of health treatment, nutrition, and hydration, is legal—as it is in almost all European countries—and has been possible since 2017 [1]. The specific conditions under which a patient may choose this option include irreversible disability or terminal illness, unbearable pain, no available care, and the patient’s informed consent. “Active euthanasia”, practiced by a physician, is considered similar to voluntary homicide in the Italian legal framework and is punishable under Articles 579 and 580 of the Criminal Code [8]. In contrast, “assisted suicide” occurs when a patient independently performs the last act that leads to death with medical assistance. If it is based on the patient’s free and conscious will, it is tolerated in certain cases. Recent Italian Constitutional Court sentences in 2019 [4] and 2025 [6] have extended the areas of non-criminal liability for “assisted suicide”, but it is not completely a right. On the contrary, in other countries next to Italian borders (for instance, Switzerland), assisted suicide is legal, and the law tolerates the practice when patients act independently and their caregivers help them without any interest in their death. For these reasons, “assisted suicide abroad” is sometimes considered by Italian patients. However, in the Italian legal framework, palliative care and the use of deep sedation with informed consent are permitted for patients in advanced stages of diseases whose symptoms are otherwise not manageable. This approach aims to relieve intractable pain by progressively reducing the patient’s consciousness until it ends.

Initially, Mrs. A. often expressed the hope of being able to turn to centers existing in another country in order to make her death as fast as possible, without pain and without existential suffering. As an ethical and emotional mediator, the psychologist started a discussion with Mrs. A. on the reasons that led her to consider this choice better for herself, focusing on the needs that she believed could be met abroad, rather than in Italy. Following end-of-life care guidelines [17], it was essential to respect her personal wishes and beliefs [9]. Such effective communication was also critical for shared decision making and for reducing adverse bereavement outcomes. This reflection continued throughout the last year of her life and accentuated in recent months, when Mrs. A. felt she had to make choices that would prevent intolerable suffering. During her meetings with the palliative care doctor and the visit to the hospice, palliative care and terminal sedation were explained to her, as recommended by current guidelines [17,18], allowing Mrs. A. to distinguish between an impulsive and desperate choice and assistance in which all her needs could be heard and accepted.

Mrs. A. was not overwhelmed by fear and despair, particularly related to painful symptoms and the anguish of death, but was able to access an experience of conscious preparation and acceptance of events with adaptive coping of adaptation to terminality and acceptance [13,14]. From that moment, suffering and the fear of the end of life took on a different connotation: Mrs. A. experienced relief and clarity of thought, and her sadness appeared to be mitigated by a sense of tranquility. During this time, the nature of her questions changed: previously, they were characterized by anger, frustration, agitation, and confusion; subsequently, they were characterized by a clearer identification of her needs and the care path she was looking for. Thereby, communication, previously fragmented and conflictual, changed. Lastly, the patient’s relatives called the psychologist to inform her of her passing. They shared their concerns about the difficulties they felt the woman had faced in managing a peaceful end of life according to her wishes. They shared her search for support to end her suffering and were relieved by the care she had received in the hospice.

The patient observed that the choice of assisted suicide, which she imagined as the first option for a painless death, was not actually respectful of factors such as the possibility of sharing and spending as much quality time as possible with her loved ones. Moreover, Mrs. A. focused on the Italian legal framework, which allows for continuous assistance from both formal and informal caregivers, and how this assistance for patients meant access to an ecological and ethical end-of-life. Mrs. A. went through this phase of her life, being able to give meaning to every moment, although difficult and painful. Thanks to the awareness achieved, the patient informed relatives and prepared them, and asked for a meeting at home in the presence of the psychologist to communicate her decisions. Hence, when a patient is able to—even helped to—communicate fears and needs, it is then necessary to start planning the choices, since they are linked to the possibility of ethical dying, as expressed by the principle of palliative oncology: “the inseparable link between relief of suffering and respect for the autonomy of terminally ill patients” [17].

Evaluating the appropriateness of this choice remains a complex process. In medical practice, a multidisciplinary assessment involving the patient is necessary to evaluate factors such as prognosis and possible treatment options. In fact (as in the case described), the patient and family must evaluate and support their choices (whether palliative care or end-of-life support), often finding themselves having to justify and explain to the medical staff a subjective existential factor, such as “existential distress” or the intolerability of their “psycho-physical” conditions. This requires the objective translation of the personal perception and sustainability of a suffering that cannot be concretely perceived or assessed by individuals, except on the basis of their own knowledge. This assessment is inevitably influenced by the ethical and moral beliefs of the people involved, be they healthcare professionals or family members.

International legislation, therefore, allows for a practical choice: a terminally ill patient has the right to access palliative care or choose physician-assisted suicide. However, this process, in reality, depends heavily on the knowledge and cultural background of the patient, who must navigate the approach of the medical team caring for them. Each healthcare provider has their own personal beliefs, and each healthcare facility has its own practices and care modalities, which, in accordance with the legislation, ultimately determine the best option for each case.

The right to refuse medical treatment is recognized in many countries, including Italy. Patients can assert their right to discontinue treatment or life support. Our reflection therefore aims to highlight this contradiction: patients can decide not to seek treatment, but there is still no clear way to make an informed choice regarding a dignified end of life.

## 5. Discussion

This case highlights the complex interplay between legal frameworks, multidisciplinary care, and patient self-determination, illustrating several implications for end-of-life practice.

Right after diagnosis, healthcare professional–patient communication is part of the care offered [17,18,19]. On the one hand, communication is typically asymmetric; on the other hand, in the end-of-life setting, this type of communication turns out to be counterproductive [20]. No one can claim to be more competent than another concerning dying, nor can one establish what is mandatory for a patient in the end-of-life stage in order to give meaning to such a condition. The final meaning of accompaniment to a so-called “good death” is possible when there is conscious acceptance [21,22]. Access to exclusive palliative care should give the patient time [23,24,25,26] to understand the transition to the end-of-life project that respects the personal characteristics of illness. This process is facilitated by empathic, respectful listening and ethically guided by the principles of the right to self-determination and to make one’s own choices in the face of end-of-life experience. This case report also suggests the importance of addressing these issues early [19], when the patient still has a good quality of life, because it is possible to do so in a situation of balance, self-determination, and possibility of choice—not in despair or urgency. Specifically, other works concur with the themes of autonomy as a determining factor of perceived dignity, the desire for control over the dying process, and the desire for self-determination. Dignity, as mediated by the loss of functionality and linked to the loss of control, and dignity as identity are themes pointed out by Mrs. A’s case [27,28].

In order to promote access to a dignified end of life, to a “good death” [29,30,31], it is necessary to set these objectives at a time when patients are still able to reason about themselves, their condition, and their principles [28], on which intervention has the greatest risks or benefits. When to intervene for a good death? As specialists in cancer and palliative care, we must remember that the human being can really gauge when it is safe [32]. In this regard, the suitable conditions are supposed to be the opportune moment [23] and the provision of properly heard and understood information [24,25].

The foundation for these conditions is the gap between “time of cure” and “care time”, in which the patient’s timing is relevant. Clinical practice typically follows relational asymmetry: time is conditioned by disease characteristics and the organizational modalities of care providers. For hospital organizations, time is “objective”; it is not specifically based on the preferences or needs of patients, but on safety criteria, which in turn are based on medical and oncological points of view. However, in palliative treatment, flexibility and openness to the patient’s wishes are mandatory in order to maintain an adequate quality of end-of-life care through the evaluation and treatment of pain and physical and psychosocial distress [9]. Healthcare professionals can be considered “active witness/participant in the suffering experience of the cancer patient and his or her loved ones” [33]. As stated by the Gestalt theorists, this “relationship provides the energy needed to develop particular adaptive abilities” [34]: it is precisely within this relationship that a multidisciplinary team helps the patient resolve doubts and reflections, restoring dignity to the patient’s feelings. From the perspective of narrative-based medicine [35], healthcare professionals’ awareness is crucial to acquire, understand, and integrate the different points of view and roles involved in the care process to build a shared and personalized path. After the expected phase of shock and reorganization [36,37], the patient and family members experience adaptation to the ongoing process, which allows them to recover an adequate and/or sufficient psycho-emotional balance, so that they return to feeling desire and hope and thus give space again to daily life, albeit conditioned by the disease and its related limitations. In particular, the psychologist’s objective is to support the patient in finding a psycho-emotional balance sufficient to go through the end-of-life process. What does this choice in the end-of-life setting consider? Given the main goal of the intervention, is it directed toward pursuing the organization’s needs or the patient’s needs? Is the team or its organization serving as a strategy, or is it becoming the goal? As healthcare professionals, we are participants in a patient’s decision-making process, because the time of death and the way of dying reflect the patient’s beliefs and choices. When a patient is supported in participating in the relationship with the healthcare team, the fundamental criterion is to make proper end-of-life choices.

## 6. Conclusions

The case report revealed differences between the time for cure, the time for care, and the time of dying. Dignity and ethics in the time of dying are possible when the time of care is granted by a multidisciplinary team. Indeed, following the situation described and considering the three critical moments above pointed out, the right time to plan the end of life becomes possible during the “care time”: within the relationship, it becomes part of a shared reflection with healthcare workers, and thanks to the support it instills, the end-of-life process becomes thinkable and the emotions connected to it can be faced [38]. At this stage, the decision-making process in the healthcare system is expected to manage the current symptoms, but each intervention has to be contextualized within a broader reflection on the meaning of such intervention. Otherwise, the experience of both specialists and users will be characterized by an increase in complexity and suffering, resulting in amplified suffering and impatience among all parties involved. This case underscores the importance of early, multidisciplinary engagement and ethical reflection in palliative care to ensure dignity and patient autonomy at the end of life.

## Data Availability

Data sharing is not applicable. No new data were created or analyzed in this study.

## Supplementary Materials

The following supporting information can be downloaded at: https://www.mdpi.com/article/10.3390/healthcare13212741/s1.

## Abbreviations

The following abbreviations are used in this manuscript:

ADI	integrated home care
