# Supplementary material for: Time of Care and Time of Dying: A Multidisciplinary Case Report on End-of-Life Experience Within the Italian Legal Framework
Source: Healthcare (Basel). 2025 Oct 29;13(21):2741. doi: 10.3390/healthcare13212741 (PMC12608887; doi:10.3390/healthcare13212741)

Supplementary Material

TIME	DIAGNOSIS	THERAPY	ASSOCIATED THERAPIES	NOTES
Since 2012 until 2019	Breast neoplasm	Neoadjuvant CT, followed by mastectomy, than hormone therapy	none	Starts therapy in Istituto Oncologico Veneto IOV IRCCS
2019-2021	peritoneal and ovarian metastasis	chemotherapy, Paclitaxel-Pertuzumab-Trastuzumab and after Perturzumab and Trastuzumab	none	from 2019 to 2021 there is a global control of disease
2021	ovarian recurrence of mammary origin and several metastatic nodules were detected	continues with ongoing therapies		
2022	peritoneal progression<br><br>Genetic analysis from which	access to experimental clinical trial	the necessary active and concurrent care treatments are	Increased fatigue and asthenia, decreased ps,

	results BRCA2+ mutation		initiated simultaneously.<br>Patient began a psychological support	drug peripheral neuropathy, and anxiety symptoms
At the end of 2022,	recovery of general condition	stop active therapies	Patient continues with individual psychological interviews and group psychotherapy	
2023	Recurrence of disease with peritoneal progression (with rcm1 colon stage IV) with	Resumed chemotherapy treatments with partial response and good tolerance.	Start supportive and palliative care.<br>Continues psychological support	Deterioration in her general condition, asthenia, and limited pain control: the woman intensified her visits with palliative care physicians
2024	Peritoneal progression of the disease	Experimental therapy was suspended and chemotherapy was resumed-	Activation of home care for clinical monitoring palliative care.<br>Continues with individual psychological interviews and	

			group psychotherapy	
2024	deterioration in her general condition: asthenia, and limited pain control. Subsequent episodes of hypotension and confusion	hospitalization in another medical ward		
2024	The oncologist explains the clinical situation, prognosis, and treatment options	continuing the current therapy	Palliative therapies are intensified	Patient evaluate with the psychologist the different options for end-of-life support
2024	After explaining the reduced chances of control of the disease	The patient decided for a new line of treatment.	Ongoing palliative therapies	She visits the hospice and is informed by the palliative care specialist about possible treatments

2025	Due to her ongoing care needs (frequent drainage, vomiting, poor nutrition)		The patient requests admission to a hospice in order to receive continuous care	
During hospitalization			The patient decided to start palliative sedation	

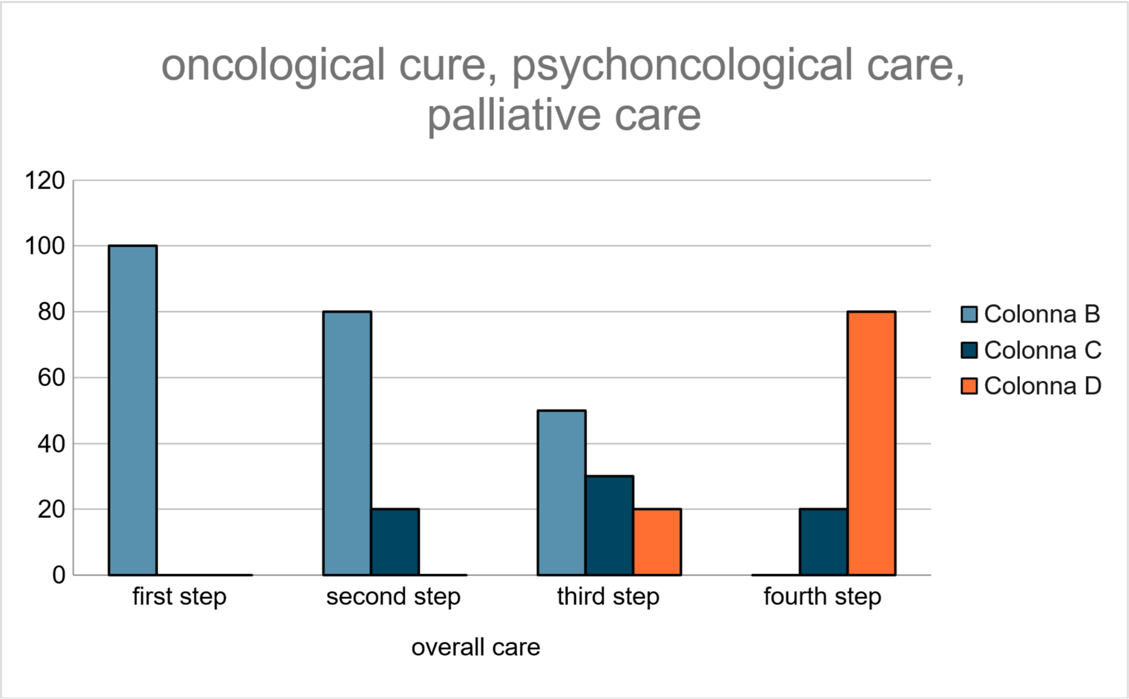

Supplement: Supplementary file 1 [file healthcare-13-02741-s001.zip › healthcare-3893086-supplementary.pdf]
